# Study protocol for evaluating Brown Buttabean Motivation (BBM): a community-based, Pacific-driven approach to health

**DOI:** 10.1186/s12889-022-12979-3

**Published:** 2022-03-31

**Authors:** Fa’asisila Savila, Warwick Bagg, Boyd Swinburn, Bert van der Werf, Dave Letele, Anele Bamber, Truely Harding, Felicity Goodyear-Smith

**Affiliations:** 1 Pacific Health, University of Auckland, Auckland, New Zealand; 2 School of Medicine, University of Auckland, Auckland, New Zealand; 3 Population Nutrition and Global Health, University of Auckland, Auckland, New Zealand; 4 Section of Epidemiology and Biostatistics, University of Auckland, Auckland, New Zealand; 5 BBM, Unit 11 613-615 Great South Road, Manukau, Auckland, New Zealand; 6 Faculty of Medical & Health Science, University of Auckland, Auckland, New Zealand; 7 Department of General Practice & Primary Health Care, General Practice & Primary Health Care, University of Auckland, PB 92129, Auckland, 1142 New Zealand

**Keywords:** Quasi-experimental design, Obesity, Physical activity, Quality of life, Weight loss, Pacific islander, Māori

## Abstract

**Background:**

Buttabean Motivation (BBM) is a Pacific-led organisation which aims to reduce obesity amongst Pacific and Māori people in New Zealand enabling them to choose a healthy and active life-style for the duration of their lives, their children, their wider family and the community. BBM offers a holistic approach to weight loss, recognising that mental health, family and cultural factors all play essential and critical role in nutrition and physical activity patterns. This study aims to evaluate the effectiveness of BBM for sustained health and wellbeing outcomes among its predominantly Pacific and Māori participants for both general BBM members and those with morbid obesity attending the ‘From the Couch’ programme.

**Methods:**

Quasi-experimental pre-post quantitative cohort study design with measured or self-reported weight at various time intervals for both cohorts. Weight will be analysed with general linear mixed model for repeated measures, and compared with a prediction model generated from the literature using a mixed method meta-analysis. The secondary outcome is change in pre- and post scores of Māori scale of health and well-being, Hua Oranga.

**Discussion:**

Multiple studies have shown that many diet and physical activity programmes can create short-term weight loss. The fundamental question is whether BBM members maintain weight loss over time. In New Zealand, Pacific and Māori engagement in health enhancing programmes remains an important strategy for achieving better health and wellbeing outcomes, and quality of life. Internationally, the collectivist cultures of indigenous and migrant and minority populations, living within dominant individualist western ideologies, have much greater burdens of obesity. If BBM members demonstrate sustained weight loss, this culturally informed community-based approach could benefit to other indigenous and migrant populations.

**Trial registration:**

Australian New Zealand Clinical Trial Registry ACTRN12621000931875 (BBM general members) First submitted 10 May 2021, registration completed 15 July 2021. ACTRN12621001676808 7 (From the Couch) First submitted 28 October 2021, registration completed 7 December 2021.

## Background

Obesity now presents the biggest health risk in Pacific populations in New Zealand. This is well documented and widely accepted. Yet despite community and government efforts to promote weight loss and encourage engagement in lifestyle change programmes, Pacific and Māori continue to have far higher rates in obesity and weight-related diseases in Aotearoa New Zealand compared with their non-Pacific, non-Māori counterparts [1]. As little as 5% weight loss for a person with obesity can lead to clinically significant reductions in several weight-related comorbidities [2]. The flow-on benefits of sustained weight loss on other risk factors such as hypertension, and hyperlipidaemia, incidence and management of diseases such as diabetes and sleep apnoea, and even mortality are well established. Too often after initial weight loss success, weight increases return and chronic illnesses persist due to shortcomings of intervention programmes, such as their short-term nature, design or costs involved, in addition to the ongoing ease of passive over-consumption in an obesogenic food environment [3]. Most existing evaluations of weight loss programmes are researcher-initiated, engage predominantly at an individual level and use stringent research designs, particularly randomised control trials. Biomedically-based interventions are often delivered by ‘experts’ to individuals or small groups without community leadership and input, and programme life expectancies can end up matching the duration of research grants.

The challenge of creating an effective programme by and for indigenous and migrant communities that successfully achieves ongoing engagement and results in sustained weight loss is still to be met [4]. A culturally-centred, community wrap-around approach is needed to inspire a sense of commitment and sustained motivation.

Buttabean Motivation (BBM) is a major Pacific-led organisation of promise that has emerged within this environment. BBM originates from the professional pseudonym ‘Brown Buttabean’ used by former boxer and founder of BBM, Dave Letele. At first glance, BBM is another physical activity programme aimed at addressing ‘the alarming obesity statistics among Māori and Pasifika’ in Aotearoa New Zealand. However, BBM offers a more holistic community-based approach, recognising that cultural factors play an essential and critical role in determining community nutrition and physical activity patterns. Physical activity is important for reducing risk of obesity, cardiovascular disease, stroke, and several other chronic diseases including type 2 diabetes, hypertension, and it impacts positively on psychological disorders (depression, anxiety), and overall quality of life [5]. Crucially, physical exercise offers a viable entry point for Pasifika and Māori on the path to wellbeing, with fitness, strength and dance being highly resonant with cultural values and practice. In the context of an obesogenic environment, sociocultural factors (norms, values and beliefs) are important in understanding and mitigating adverse social determinants on health [6].

BBM transcends conventional western definitions and parameters of weight loss programmes, emphasising a Pacific-centric philosophy in relation to family and community, rather than, highlighting individual aims in achieving personal health or a desired body aesthetic. Pacific and Māori cultural values and beliefs are demonstrated in the programme’s wider social support initiatives, such as pop-up health clinics and community education, finding innovative ways to motivate participants to adopt wider lifestyle actions around diet and other health behaviours. This philosophy is also visible in more fundamental forms of social support offered, such as community housing and a food bank, which are well-established factors influencing nutrition-related health outcomes. Very few existing weight loss programmes employ a holistic community focussed approach. Despite the challenges of evaluating evolving, holistic programmes, especially with the disruptions caused by Covid-19 impacts on people’s lives and social interactions, it is important to answer research questions of effectiveness and impact of such community-led programmes [7].

BBM was set up in 2014 and, by September 2021, there were over 15,000 members who variably participated in several programmes including 34 weekly free community bootcamps. BBM also offers a 12-week ‘From the Couch’ (FTC) programme, which is a highly supportive, holistic, physical activity and tailored diet programme designed for individuals who are usually morbidly obese and have very restricted physical capacity, such as not being able to ‘get off the couch or who struggle to stand for very long periods of time’. The FTC programme aims to enable individuals to move ‘from the couch’ towards eventually being able to do everyday activities, which most able-bodied people take for granted.

The BBM programme warrants a full evaluation not only to measure its social and health impacts, but to understand its internal dynamics, values and strengths to inform continuous programme improvement and increase reach and effectiveness [8]. A preliminary qualitative study using the Fonofale Pacific framework of inquiry [9] has explored the lived experience of Pacific and Māori clients and trainers. A further study is underway to conduct a process evaluation of BBM’s community engagement through its in-person, social and news media outreach activities with respect to the health and wellbeing of Pasifika and Māori people in their community over time. The underlying sociocultural values that determine sustained motivation, engagement and impact, and how these are associated with biomedical outcomes needed to be researched.

### Aim and objectives

The aim of this study is to evaluate the effectiveness of BBM for sustained health and wellbeing outcomes among its predominantly Pacific and Māori participants. The primary hypothesis is that BBM will create valuable, average sustained weight loss (≥ 5% of baseline weight) for participants over three years. Secondary hypotheses include that programme participation will result in improved quality of life (measured in Hua Oranga); and for the FTC cohort, improved cardiovascular and metabolic health outcomes (measured via medical records).

There are two research questions. The first is, how effective are BBM’s general programmes in providing sustained weight loss of ≥ 5% of baseline body weight and improved quality of life measures for its members? The objective is to carry out an impact assessment of BBM’s general programme effectiveness, to estimate the medium to long-term (one to two year) weight loss maintenance and the impact on quality of life of BBM members.

The second question is how effective is BBM’s FTC programme for achieving sustained clinically significant benefits for members who typically experience of Class 3 or 4 obesity [10]? The objective here is an outcome assessment of the FTC programme’s effectiveness, to examine the medium to long-term clinical impact on a sub-cohort (n ~ 50) of members who enter BBM with Class 3 and 4 obesity, body mass index (BMI) > 35 kg/m^2^. As secondary outcomes, we will collect laboratory and clinical data from medical records held by primary care providers. Data collection will occur on three occasions, at commencement of the FTC programme, one and two years later.

## Methods/design

### Study design

This is a quasi-experimental quantitative cohort study design. The general programmes participants will have pre-measurements (measured or self-reported weight at entry to BBM) and multiple post-measurements (measured weight at various intervals), and the FTC participants will have pre-post programme weight measurements. Evidence from the literature of the natural course of weight change in populations like the BBM participants with and without interventions will enable counterfactual comparisons. This study follows the Strengthening the Reporting of Observational Studies in Epidemiology (STROBE) Statement and a Standard Protocol Items: Recommendations for Intervention Trials (SPIRIT) checklist has been completed.

### Setting

Attendees of BBM programmes in Auckland from 2021.

### Participant recruitment

For the general programme, participants will be recruited through study advertisements on BBM’s social media platforms, and as posters in physical locations (the exercise centres). BBM trainers will promote the study to BBM attendees. All attendees over the age of 16 years will be eligible to participate.

For FTC group participants, all people registering for three 12-week programmes will be invited to enrol in the study. All FTC participants will be eligible, with the exclusion of repeat attenders already enrolled in the study.

Participants from both the general and FTC groups will be offered a koha (gift) of a $40 token at the 24-month follow-up. Participants will continue to be enrolled until the target samples are reached. Given recurrent lockdowns and restrictions on class sizes due to COVID-19, it is anticipated that the study will be extended by one year to enable 24 month follow-up of all participants.

### Sample size calculations

The study has a finite population and has a quasi-experimental design of an existing intervention with no randomised sample, hence our sample size needs to take a pragmatic approach. We will recruit a many eligible participants as possible in the available timeFor the general group the target is to recruit 1000 BBM members, and for FTC. there are about 20 enrolees in each FTC programme, and some repeat the cycle, so the total number is estimated to be about 50 over three cycles.

### Variables

For the general programme group, enrolment measurements will be height in metres and weight in kilograms (kg), and they will complete a Māori scale of health and well-being, Hua oranga, electronically. Their weight in kg will be recorded at six and 12 months, and at conclusion at 24 months, they will repeat Hua oranga and have a final weight taken. The primary outcome will be changes in weight, and the secondary outcome will be changes in Hua oranga scores. Note that first measurement is at enrolment in the evaluation and many participants at this stage will have been in the BBM programme for a period of time already. At enrolment, their self-reported heaviest weight, and weight at their first BBM visit will be recorded.

For the FTC group, in addition to the data collected above, clinical data from their health records will be sought. This includes diagnosis classifications, past history and current problems of any chronic diseases, especially type 2 diabetes, hypertension, gout, dyslipidaemia, cardiovascular disease, stroke, renal dysfunction, liver disease, osteoarthritis, gall bladder disease, chronic obstructive respiratory disease, and obstructive sleep apnoea. Blood and urine analyses laboratory results, prescribed medications, and other referral letters and discharge summaries will also be accessed. Their blood pressure at enrolment and at 12-weeks will also be recorded.

### Data collection

Pacific research assistants will undergo training in recruitment and data collection and be provided with detailed step-by-step instructions. They will contact respondents to arrange a time and location to review the Participant Information Sheet (PIS) and gain consent, preferably at a BBM exercise centre. If requested, the PIS and consent form can be provided in te reo (Māori language), Samoan and Tongan. A log of all attempted communications with potential participants will be maintained. Once participants have consented, they will be invited to complete the online survey, which includes length of time since first joining BBM, weight at that time, frequency of attendance at BBM exercise sessions in the last three months, the Hua Oranga survey and demographic details (such as age, gender, ethnicity).

All participants will have their height and weight measured at enrolment by BBM staff and research assistants who will have undergone training using standardised protocols [11]. Participants will be weighed using Seca Scale Column Model 703 300 kg scales without footwear and light clothing only. Height is measured using the study’s stadiometer without shoes. Participants’ retrospective self-recorded weights, for example when they first entered the BBM programme, will also be collected.

Additional data will be collected for FTC participants. BBM trainers already measure and record blood pressure in this group, using an extra-large cuff calibrated sphygmomanometer. All FTC participants will be asked to consent to accessing their National Health Identification number and/or details of their primary healthcare provider / general practitioner to enable extraction of clinical details from their medical records: chronic disease diagnoses, diagnostic laboratory results and reports, clinic letters, e-referrals, prescribed medications, hospital appointments and admissions, and discharge summaries. Most New Zealand patients are enrolled in a Primary Health Organisation (PHO) through their registered general practice. PHOs collect primary care data from their member practices [12]. There are eight geographically dispersed and overlapping PHOs in the Auckland region. It is anticipated that most FTC participants will be enrolled with the three largest PHOs operating in South Auckland (where most BBM members live). Patient data will be extracted by these organisations on application using their data management processes. Where participants attend practices that belong to other PHOs, the clinical data will be sought via direct contact with individual practices.

Routine data for FTC participants collected by BBM will be included in the analyses. These data include age, frequency of exercise per week, whether they suffer from a respiratory condition or asthma, high blood pressure, blood disorder, diabetes, arthritis, epilepsy of seizures, past history of stroke, and whether they have been pregnant, had major surgery or hospitalised in the previous 12 months.

### Statistical analysis

All statistical analysis will be done in R [13] using the lme4 package [14]. The primary outcome of interest is sustained weight loss. How successful BBM is at helping participants lose and maintain 5% weight loss will be a critical outcome measure, however, the effectiveness of BBM compared to other counterfactuals will also be assessed.

The general programme cohort will include existing and new entrants. For existing members we will also have self-reported weights at their first BBM session, which will be considered the baseline or ‘time zero (T0)’ weight with subsequent times measured from then. For example, for a person who started at BBM seven months before enrolment in the study, their reported weight at BBM entry will be T0, their enrolment weight (measured by the research team at study enrolment) will be T7, their 6-month weight will be T13, 24-month weight will be T31. Typically, the pattern of weight loss with weight loss programmes is tick-shaped with a weight loss nadir at 6–12 months followed slow return back towards T0 weight or beyond. Estimated duration of the study will be 24 months of follow-up from enrolment.

### Determination of the counterfactuals for comparison

Since there is no enrolled comparison group in this study, the counterfactuals to be included to determine how effective BBM is will be threefold:

1. Versus similar New Zealand population. The estimated mean weight change of ethnic/gender/age groups will be derived from the last five years of NZ Health Surveys [15] and these will be compared with the general programme groups participating in BBM. Note that, longitudinally, weight change in a subpopulation is the additive effects of secular changes to weight (ie each age group gets a little heavier each year) and age-related weight change (ie the person moves to an older age group which has a higher average weight).
2. Versus control groups in other similar studies. A literature review [16] has identified five weight loss studies with predominantly Pacific or Māori participants that had control groups (total *N* = 955, follow-up 3–24 months) [17–21]. Their weight change trajectories will be compared to the BBM general programme participants.
3. Versus other interventions. The literature review noted above [16] identified 18 studies with various interventions with weight change outcomes [17–34]. These combined results show how effective other programmes have been. Since the duration of interventions varies (3–24 months), weight loss is not linear.

**Fig. 1:**
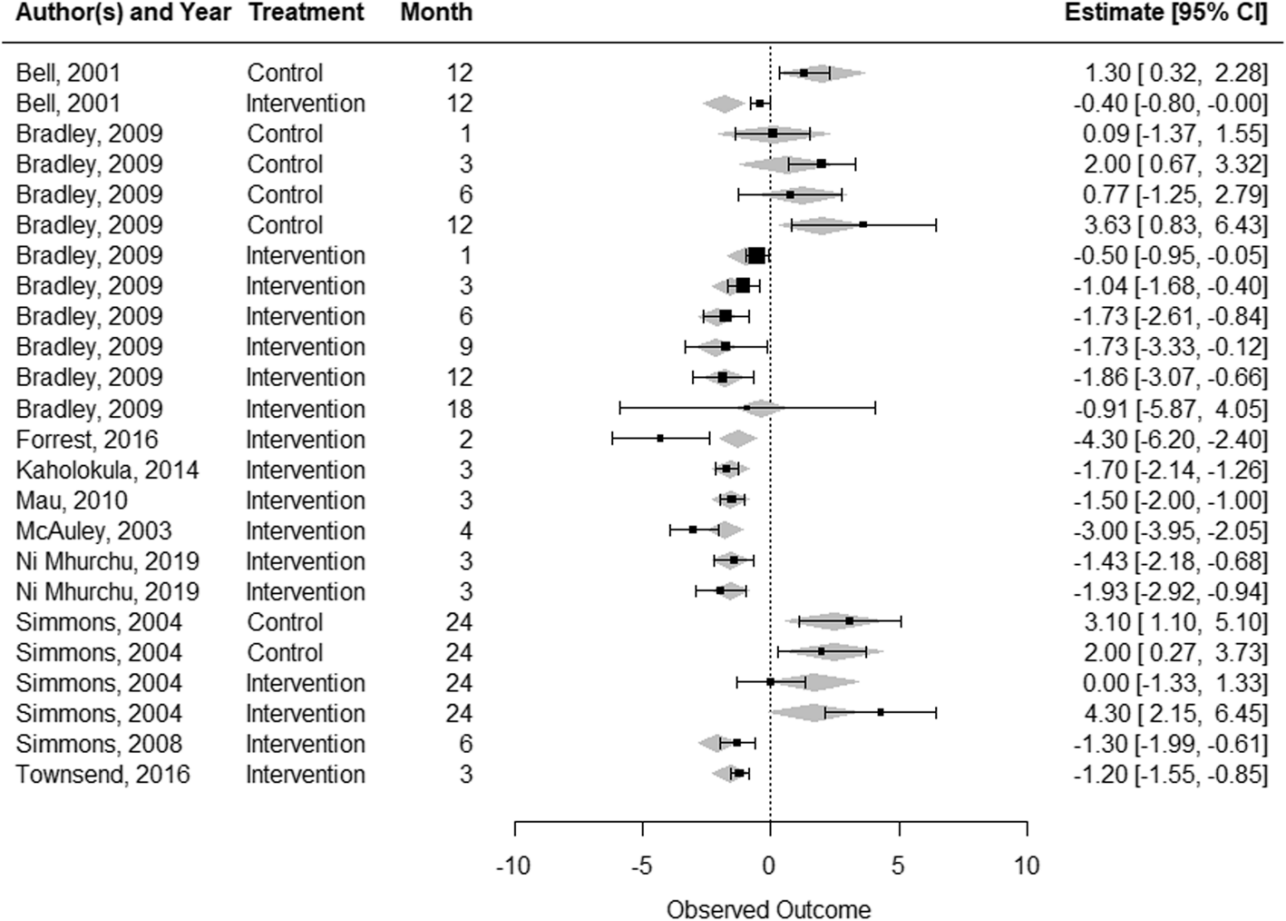
Forest plot of effect size and confidence intervals for controls and interventions in the included studies

A mixed method meta-analysis was conducted using the metaphor package [35] in R. Studies were only included in this analysis where the difference with baseline (time 0) was published together with their standard error, standard deviation, or confidence interval (to enable calculation of the standard error). Figure 1 shows the forest plot of the included studies.

**Fig. 2:**
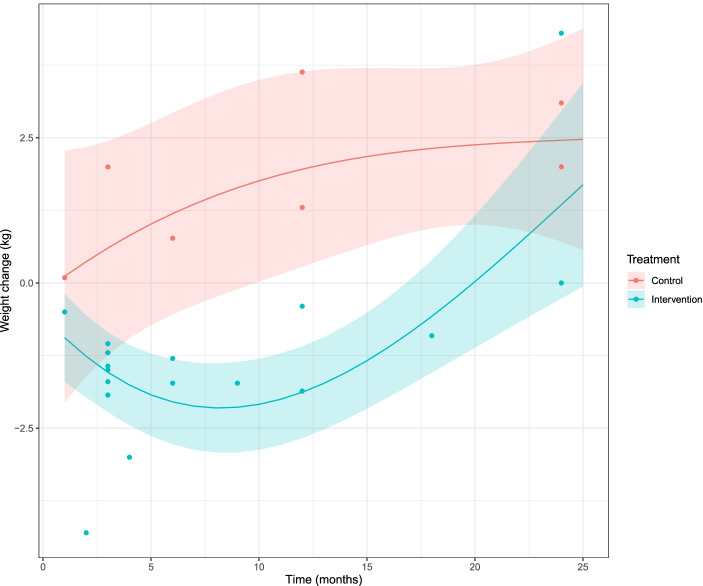
Weight change in kilograms against time in months for each study in the meta-analysis

The moderator variables were intervention, time, and interaction intervention and time, and the response variable was the weight change in time from a baseline of zero for both control and intervention. The random variable was the interaction between the individual studies and the intervention. Spline functions are formed by joining polynomials together at fixed points called knots*.* The number and position of knots in the spline function was found by evaluating the Akaike Information Criterium (AIC) using all possible combinations of placement of two knots [36]. The best AIC was found with one knot at two months. Splines were compared with polynomials, but the fit of a spline function was superior to them. A spline function with one knot could be fitted within a mixed effects meta-analysis to describe the initial weight loss followed by increase in weight (Fig. 2).

**Fig. 3:**
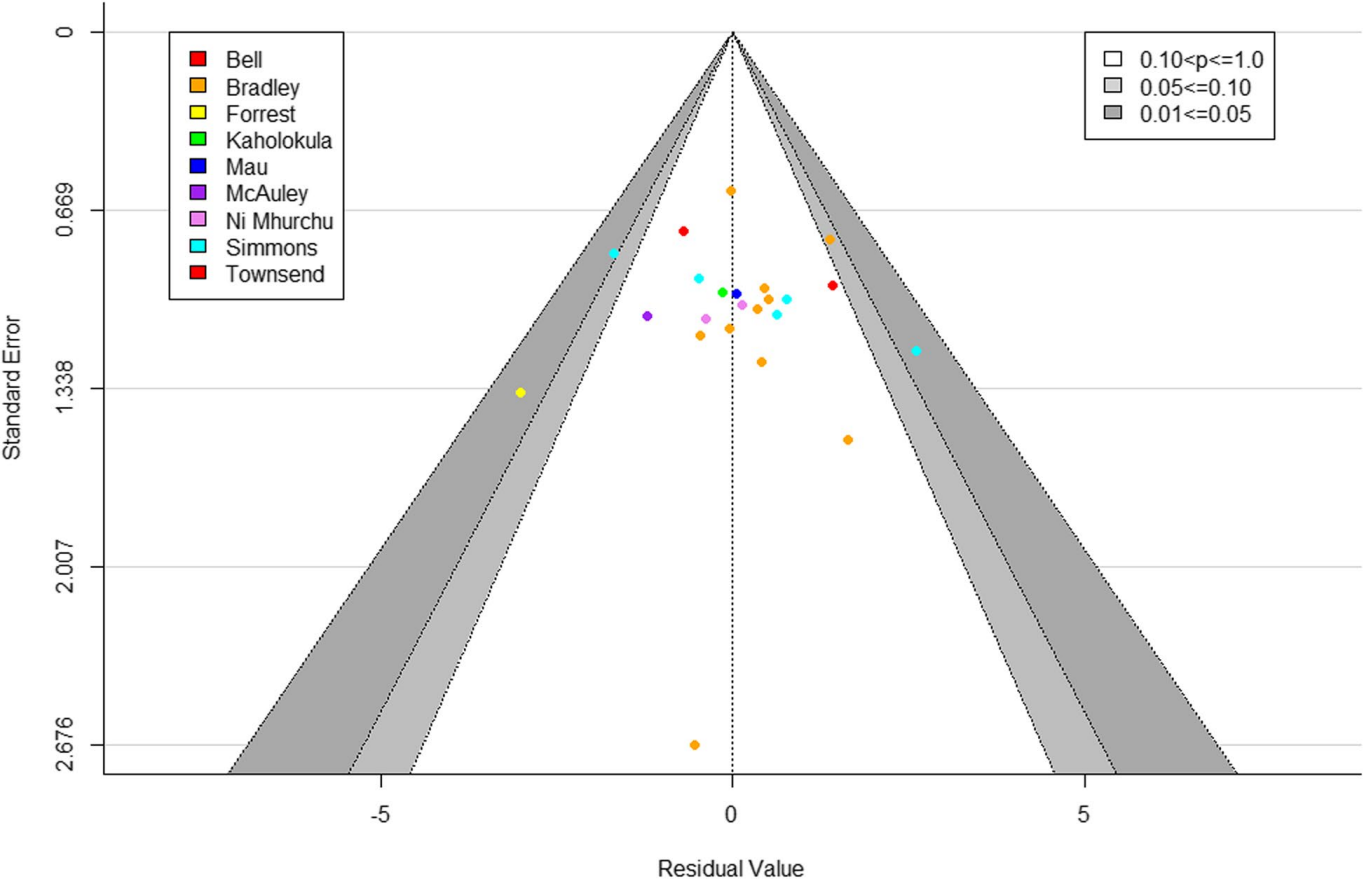
Forest plot of the standard errors from the studies used in the meta-analysis

A funnel plot of the standard errors against the residual values after fit was symmetrical, with a non-significant Eggers test (*p* = 0.8505), indicating no evidence of publication bias in the articles used in the meta-analysis (Fig. 3).

Weight for both the general BBM and the FTC cohorts will be analysed as with a general linear mixed model for repeated measures, with person (participant) as the random variable and time as the fixed variable. The two cohorts will be combined because some general BBM members may previously have been FTC participants, and some FTC participants subsequently become general members. The weights will be logarithmic transformed before analysis, if the residual analysis indicates that. The comparison of our study data with the tick shaped curve prediction model generated from the literature (Fig. 2) will be done by comparing specific points in time and calculating the t-probability of being equal on basis of their 95% confidence intervals.

The FTC group will be considered as a series of three cohorts of about 20 participants each entering the FTC programme. An estimate of 80% recruitment into the study will give 50 participants who will have baseline and follow up data. There is also no parallel comparison group for this part of the study. However, their weight change trajectories can also be compared with the counterfactuals noted above. FTC data will be analysed with a Difference in Difference (DID) analysis using the estimated covariance matrix from the repeated measures analysis to obtain the corrected differences (Treatment – Baseline) – (Control – Baseline) as an estimate of the effect of the programme.

### Analysis of hua oranga scores

The hua oranga scores will be analysed with a general or generalised linear mixed model with proper distribution (gaussian, binomial or poisson) depending on the characteristics of the distribution of the outcomes. The random term is the person (participant). The scores per domain will be analysed as well as the sum of all the scores. Because we have pre- and post-data, all analyses will be followed with a DID analysis to obtain the corrected differences.

### Data management

All data will be stored securely on a password-protected shared drive on a University of Auckland with access restricted to the relevant researchers conducting the data collection and analysis.

### Data monitoring

As this is not an intervention study but observation of an existing programme, there is no need for a data monitoring committee nor strategies to stop a trial. Participants can freely chose when they wish to engage with BBM and repeated measurements of their weight and quality of life at the beginning and end of the study has low risk of harm.

### Dissemination

The findings will be published in appropriate peer-reviewed publications, as well as disseminated through community fono and hui (meetings) and conferences. BBM will further disseminate the findings via their social network platforms to BBM members and followers. All researchers who meet the criteria will be authors and no professional writers will be used.

## Discussion

It is important to note that the fundamental question for both our study groups is whether weight loss can be maintained over the long haul. Multiple studies have shown that many diet and physical activity programmes can create short-term weight loss. Similarly, many studies have shown that the loss and maintenance of weight can have important health benefits. The critical remaining questions are implementation-related: How many people can be attracted to a programme (especially from communities with high obesity prevalence), how many can stay with the programme (duration of engagement is closely related to weight loss maintenance), and what are the long-term weight loss results?

If this study demonstrates robust empirical evidence of programme success, this could shift the paradigm for achieving the ultimate public health objective of sustained weight loss in an era of high sedentariness and energy-dense food environment. This paradigm switch, especially for communities with a collectivist culture, may result in a move from the one-on-one advice of expert professionals in a clinic environment, to a many-on-many participation with non-experts in community settings. Internationally, it is the collectivist cultures of indigenous and migrant and minority populations, living within dominant individualist western ideologies, which have much greater burdens of obesity [37]. If a robust and culturally informed evaluation of BBM demonstrates the effective features of this community-based approach to sustained weight loss, this could be of high relevance and benefit to other indigenous and migrant populations. In Aotearoa, achieving equity for Pacific and Māori engagement in health enhancing programmes remains an important strategy for achieving better health and wellbeing outcomes, and quality of life for whānau (family).

Obesity is the consequence of complex, adaptive, societal systems and therefore approaches to deal with obesity (treatment or prevention) need to use systems tools to manage that complexity [8]. There is a lack of evidence implementing systems-level thinking and analyses to evaluate and assist in the evolution of a community programme delivering health and wellbeing services for Pacific and Māori people which uses culturally appropriate methods of inquiry [38]. The partnership of BBM and University of Auckland researchers are exploring the evidence base for BBM using kaupapa Māori and co-design [39], with the aim of improving health outcomes and reducing health inequities [40, 41]. The Pacific approaches of fa’afaletui [42, 43] and fonofale [44], and the similar te whare tapa whā model [45] use an empowerment rather than a deficit model, with a holistic view of physical, mental, spiritual and family / community health in the context of people’s lives.

Our participants are willing partakers of the BBM programme, who have joined to make a change in their health status and lives going forward. Their motivation to engage in research that aims to improve health and wellbeing has been demonstrated in our prior qualitative investigation. Combining indigenous cultural frameworks in the delivery of health support services may prove critical for sustained engagement, and significantly improved outcomes for Pacific and Māori populations.

While a pragmatic before-after study design has no control group, there is very strong evidence that a non-intervention counterfactual of no weight change is plausible, since the average natural trajectory of people with obesity is no change or slow increases in weight. A sustained weight loss of 5–10% is clinically meaningful for health benefits, even if people remain overweight or even obese [2].

Inevitably, the COVID-19 pandemic adds additional challenges. Recurrent Alert Level 4 and 3 lockdowns, the latest in August to October 2021, has delayed initial recruitment. It may be several months before the city is again at Alert Level 1 and full social gatherings can be resumed, including BBM bootcamps. In the interim, BBM provides online classes via their social media platform, as well as distributing hundreds of parcels of donated healthy food daily to vulnerable and socioeconomically disadvantaged members experiencing food insecurity through the financial stresses imposed by the lockdown.

## Data Availability

Not applicable.

## Abbreviations

BBM: Brown Buttabean Motivation
BMI: Body mass index
FTC: From The Couch
GP: General practitioner
PHO: Primary Health Organisation
PIS: Participant Information Sheet
